# Bis(2-aminomethyl-1*H*-benzimidazole-κ^2^ *N* ^2^,*N* ^3^)bis­(nitrato-κ*O*)copper(II)

**DOI:** 10.1107/S1600536812020910

**Published:** 2012-05-16

**Authors:** Jing Zhao, Heng Zhang, Xueliang Zhai, Guoyi Zhu

**Affiliations:** a Changchun Institute of Applied Chemistry, Chinese Academy of Sciences, Changchun 130022, People’s Republic of China; b Graduate University of Chinese Academy of Sciences, Beijing 100049, People’s Republic of China; c Instrumental Analysis Center, Hebei Normal University, Shijiazhuang 050024, People’s Republic of China

## Abstract

In the title compound, [Cu(NO_3_)_2_(C_8_H_9_N_3_)_2_], the Cu^II^ atom, lying on an inversion center, has a distorted octa­hedral coordination environment defined by four N atoms from two chelating 2-amino­methyl-1*H*-benzimidazole ligands and two O atoms from two monodentate nitrate anions. In the crystal, N—H⋯O hydrogen bonds link the complex mol­ecules into a three-dimensional network. An intra­molecular N—H⋯O hydrogen bond is also observed.

## Related literature

For the synthesis of the 2-(2-amino­meth­yl)benzimidazole ligand, see: Pascaly *et al.* (2001 ▶). For the structures and properties of transition metal complexes with 2-(2-amino­meth­yl)benzimidazole ligands, see: Gable *et al.* (1996 ▶); Gómez-Segura *et al.* (2006 ▶); He *et al.* (2003 ▶); Jiang *et al.* (2004 ▶).
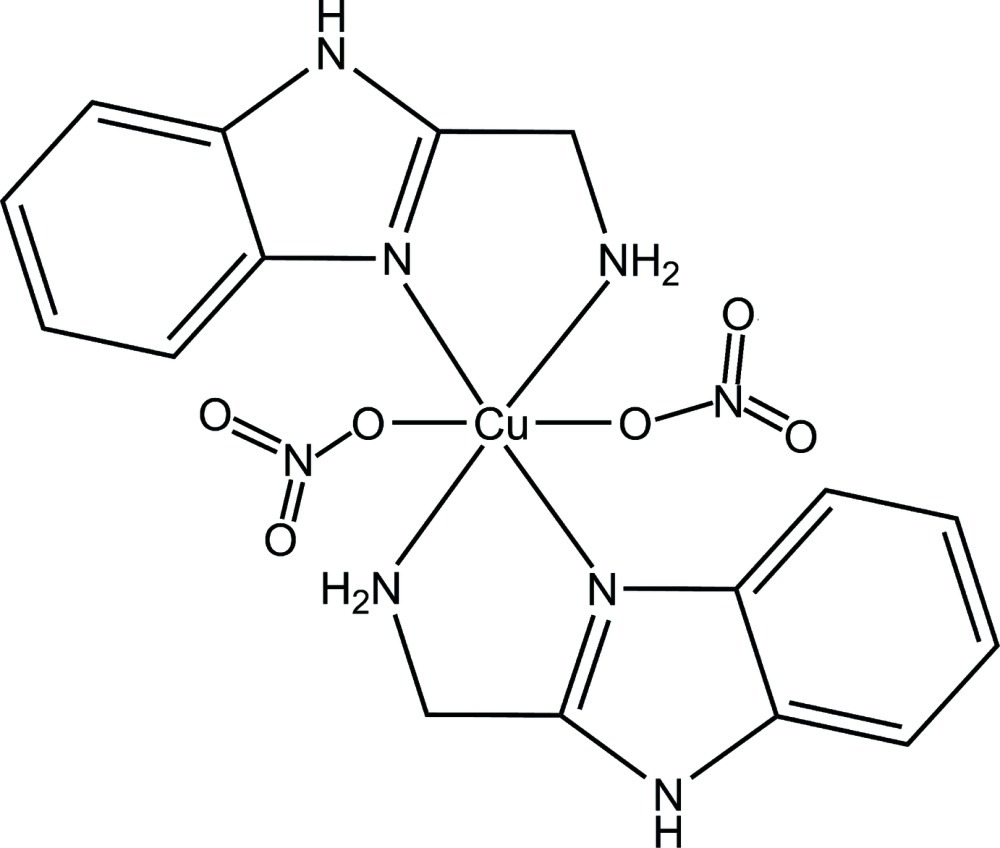


## Experimental

### 

#### Crystal data

[Cu(NO_3_)_2_(C_8_H_9_N_3_)_2_]
*M* *_r_* = 481.93
Trigonal, 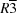

*a* = 24.6913 (8) Å
*c* = 7.9620 (5) Å
*V* = 4203.8 (4) Å^3^
*Z* = 9
Mo *K*α radiation
μ = 1.23 mm^−1^
*T* = 184 K
0.34 × 0.21 × 0.11 mm

#### Data collection

Bruker APEXII CCD diffractometer
Absorption correction: multi-scan (*SADABS*; Sheldrick, 1996 ▶) *T*_min_ = 0.681, *T*_max_ = 0.877
7196 measured reflections
1833 independent reflections
1764 reflections with *I* > 2σ(*I*)
*R*_int_ = 0.015

#### Refinement

*R*[*F* ^2^ > 2σ(*F* ^2^)] = 0.023
*wR*(*F* ^2^) = 0.063
*S* = 1.04
1833 reflections
142 parameters
H-atom parameters constrained
Δρ_max_ = 0.52 e Å^−3^
Δρ_min_ = −0.23 e Å^−3^

### 

Data collection: *APEX2* (Bruker, 2007 ▶); cell refinement: *SAINT* (Bruker, 2007 ▶); data reduction: *SAINT*; program(s) used to solve structure: *SHELXS97* (Sheldrick, 2008 ▶); program(s) used to refine structure: *SHELXL97* (Sheldrick, 2008 ▶); molecular graphics: *SHELXTL* (Sheldrick, 2008 ▶); software used to prepare material for publication: *SHELXTL*.

## Supplementary Material

Crystal structure: contains datablock(s) I, global. DOI: 10.1107/S1600536812020910/hy2548sup1.cif

Structure factors: contains datablock(s) I. DOI: 10.1107/S1600536812020910/hy2548Isup2.hkl

Additional supplementary materials:  crystallographic information; 3D view; checkCIF report

## Figures and Tables

**Table 1:** Hydrogen-bond geometry (Å, °)

*D*—H⋯*A*	*D*—H	H⋯*A*	*D*⋯*A*	*D*—H⋯*A*
N3—H3*A*⋯O1^i^	0.92	2.42	3.266 (2)	153
N3—H3*A*⋯O3^i^	0.92	2.37	3.0521 (17)	131
N3—H3*B*⋯O1^ii^	0.92	2.33	3.1036 (19)	142
N2—H2⋯O3^iii^	0.88	2.50	3.0276 (18)	119
N2—H2⋯O3^iv^	0.88	2.40	3.0823 (18)	134
N2—H2⋯O2^iii^	0.88	2.20	2.8901 (17)	135

Symmetry codes: (i) 
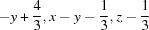
; (ii) 
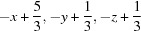
; (iii) 
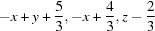
; (iv) 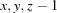.

**Fig. 1.**
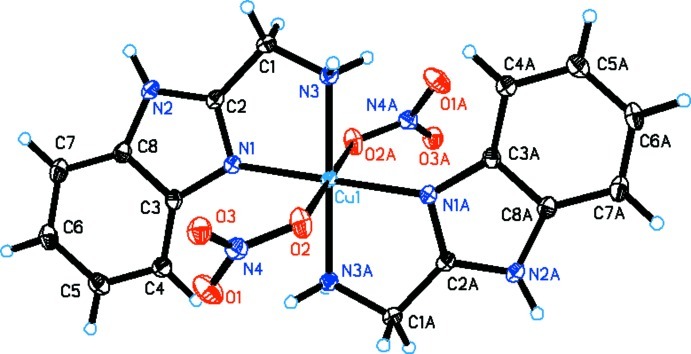
Molecular structure of the title complex, with displacement ellipsoids drawn at the 30% probability level. [Symmetry code: (A) -x+5/3, -y+1/3, -z+1/3.]

**Fig. 2.**
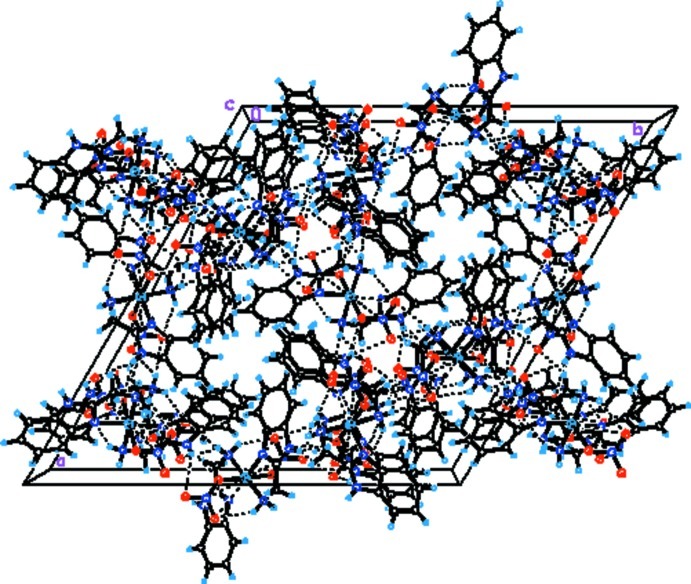
The packing diagram viewed along the c axis.

## supplementary crystallographic information

### Comment

Benzimidazole is of considerable interest as a ligand for transition metal ions. Some of their polyfunctional derivatives have been proved to possess extensive biological activities (Gómez-Segura *et al.*, 2006). Therefore, substituted benzimidazoles have attracted interest of various research groups, especially the substitution at 1, 2 and 5 positions of the benzimidazole ring is very important for their coordination behavior. The 2-(2-aminomethyl)-1*H*-benzimidazole (AMBI) ligand is a suitable model system for compounds of this sort, which is a bidentate ligand and can chelate a 3d metal ion through two nitrogen atoms of the pendant aminomethyl group and the imidazole ring (Gable *et al.*, 1996; He *et al.*, 2003; Jiang *et al.*, 2004). Moreover, AMBI possesses a larger conjugated π-system and a nitrogen electron-donor of the secondary amine group, which has an important effect on the structures and functions of the complexes. On the other hand, metalloproteins that contain Cu are widespread. Characterization of model Cu complexes that mimic Cu proteins has led to a better understanding of the chemistry of Cu in biological systems. A new copper(II) complex with AMBI, which is reported in this paper, may be of interest with respect to both of the above-mentioned areas.

In the title compound, as shown in Fig. 1, two bidentate AMBI ligands are coordinated to the Cu^II^ atom *via* two N atoms and two nitrate anions are coordinated to the Cu^II^ atom *via* one O atom. The coordination geometry around the Cu^II^ atom, which lies on an inversion center, is distorted octahedral, with a bite angle of 83.84 (5)° for two bidentate ligands. The other *cis* bond angles at the Cu^II^ atom fall in the range of 80.71 (5)–99.29 (5)° and the *trans* bond angles are 180°, suggesting a significant deviation from a perfect octahedral coordination. The Cu—N bond lengths are 1.9839 (12) and 2.0244 (12) Å, with an average of 2.0042 (12) Å. The Cu—O bond length is 2.5870 (12) Å. Extensive N—H···O hydrogen bonds in the crystal, as shown in Fig. 2 and Table 1, link the complex molecules into a three-dimensional network.

### Experimental

The title compound was prepared by adding a methanol-water solution (4:1 *v*/*v*, 5 ml) of Cu(NO_3_)_2_.3H_2_O (0.1 mmol) to a methanol solution (5 ml) of 2-(2-aminomethyl)benzimidazole (0.2 mmol) (Pascaly *et al.*, 2001). The blue mixture was stirred at room temperature for 4 h and then filtered. Purple crystals suitable for X-ray diffraction were obtained by slow evaporation of the solvent after several days. Analysis, calculated for C_16_H_18_CuN_8_O_6_: C 39.88, H 3.76, N 23.25%; found: C 39.92, H 3.75, N 23.30%.

### Refinement

H atoms bonded to C and N atoms were positioned geometrically and refined as riding atoms, with C—H = 0.95 (aromatic), 0.99 (CH_2_) and N—H = 0.88 (NH), 0.92 (NH_2_) Å and with *U*_iso_(H) = 1.2*U*_eq_(C,N).

### Crystal data

**Table:**

[Cu(NO_3_)_2_(C_8_H_9_N_3_)_2_]	*D*_x_ = 1.713 Mg m^−^^3^
*M**_r_* = 481.93	Mo *K*α radiation, λ = 0.71073 Å
Trigonal, *R*3	Cell parameters from 5478 reflections
Hall symbol: -R 3	θ = 2.7–26.0°
*a* = 24.6913 (8) Å	µ = 1.23 mm^−^^1^
*c* = 7.9620 (5) Å	*T* = 184 K
*V* = 4203.8 (4) Å^3^	Block, purple
*Z* = 9	0.34 × 0.21 × 0.11 mm
*F*(000) = 2223	

### Data collection

**Table:**

Bruker APEXII CCD diffractometer	1833 independent reflections
Radiation source: fine-focus sealed tube	1764 reflections with *I* > 2σ(*I*)
Graphite monochromator	*R*_int_ = 0.015
φ and ω scans	θ_max_ = 26.0°, θ_min_ = 1.7°
Absorption correction: multi-scan (*SADABS*; Sheldrick, 1996)	*h* = −27→30
*T*_min_ = 0.681, *T*_max_ = 0.877	*k* = −25→30
7196 measured reflections	*l* = −9→8

### Refinement

**Table:**

Refinement on *F*^2^	Primary atom site location: structure-invariant direct methods
Least-squares matrix: full	Secondary atom site location: difference Fourier map
*R*[*F*^2^ > 2σ(*F*^2^)] = 0.023	Hydrogen site location: inferred from neighbouring sites
*wR*(*F*^2^) = 0.063	H-atom parameters constrained
*S* = 1.04	*w* = 1/[σ^2^(*F*_o_^2^) + (0.0358*P*)^2^ + 6.4022*P*] where *P* = (*F*_o_^2^ + 2*F*_c_^2^)/3
1833 reflections	(Δ/σ)_max_ = 0.001
142 parameters	Δρ_max_ = 0.52 e Å^−^^3^
0 restraints	Δρ_min_ = −0.23 e Å^−^^3^

### Special details

**Table:**

Geometry. All e.s.d.'s (except the e.s.d. in the dihedral angle between two l.s. planes) are estimated using the full covariance matrix. The cell e.s.d.'s are taken into account individually in the estimation of e.s.d.'s in distances, angles and torsion angles; correlations between e.s.d.'s in cell parameters are only used when they are defined by crystal symmetry. An approximate (isotropic) treatment of cell e.s.d.'s is used for estimating e.s.d.'s involving l.s. planes.
Refinement. Refinement of *F*^2^ against ALL reflections. The weighted *R*-factor *wR* and goodness of fit *S* are based on *F*^2^, conventional *R*-factors *R* are based on *F*, with *F* set to zero for negative *F*^2^. The threshold expression of *F*^2^ > σ(*F*^2^) is used only for calculating *R*-factors(gt) *etc*. and is not relevant to the choice of reflections for refinement. *R*-factors based on *F*^2^ are statistically about twice as large as those based on *F*, and *R*- factors based on ALL data will be even larger.

### Fractional atomic coordinates and isotropic or equivalent isotropic displacement parameters (Å^2^)

**Table:**

	*x*	*y*	*z*	*U*_iso_*/*U*_eq_	
Cu1	0.8333	0.1667	0.1667	0.01942 (10)	
O1	0.85026 (6)	0.28108 (7)	0.46236 (18)	0.0427 (3)	
O2	0.89291 (6)	0.22280 (5)	0.43740 (16)	0.0339 (3)	
O3	0.94763 (5)	0.31844 (5)	0.52565 (15)	0.0321 (3)	
N1	0.84262 (6)	0.24335 (6)	0.05919 (15)	0.0205 (3)	
N2	0.89615 (6)	0.31981 (6)	−0.12266 (16)	0.0241 (3)	
H2	0.9235	0.3420	−0.2009	0.029*	
N3	0.90693 (6)	0.18332 (6)	0.01943 (15)	0.0222 (3)	
H3A	0.9417	0.1958	0.0856	0.027*	
H3B	0.8986	0.1469	−0.0339	0.027*	
N4	0.89671 (6)	0.27418 (6)	0.47583 (16)	0.0249 (3)	
C1	0.92024 (7)	0.23207 (7)	−0.10792 (19)	0.0237 (3)	
H1A	0.9053	0.2126	−0.2196	0.028*	
H1B	0.9658	0.2615	−0.1149	0.028*	
C2	0.88719 (7)	0.26609 (7)	−0.05640 (18)	0.0207 (3)	
C3	0.82031 (7)	0.28550 (7)	0.06771 (18)	0.0212 (3)	
C4	0.77224 (8)	0.28515 (8)	0.1601 (2)	0.0283 (3)	
H4	0.7479	0.2524	0.2367	0.034*	
C5	0.76134 (8)	0.33433 (8)	0.1360 (2)	0.0326 (4)	
H5	0.7284	0.3348	0.1967	0.039*	
C6	0.79714 (9)	0.38357 (8)	0.0253 (2)	0.0349 (4)	
H6	0.7886	0.4169	0.0140	0.042*	
C7	0.84461 (8)	0.38431 (8)	−0.0675 (2)	0.0319 (4)	
H7	0.8693	0.4176	−0.1426	0.038*	
C8	0.85467 (7)	0.33414 (7)	−0.04615 (18)	0.0232 (3)	

### Atomic displacement parameters (Å^2^)

**Table:**

	*U*^11^	*U*^22^	*U*^33^	*U*^12^	*U*^13^	*U*^23^
Cu1	0.01800 (15)	0.01646 (14)	0.02290 (16)	0.00795 (10)	0.00401 (9)	0.00463 (9)
O1	0.0359 (7)	0.0535 (8)	0.0510 (8)	0.0317 (7)	−0.0132 (6)	−0.0165 (6)
O2	0.0334 (6)	0.0253 (6)	0.0454 (7)	0.0165 (5)	−0.0137 (5)	−0.0077 (5)
O3	0.0276 (6)	0.0252 (6)	0.0372 (7)	0.0086 (5)	−0.0043 (5)	−0.0033 (5)
N1	0.0208 (6)	0.0184 (6)	0.0215 (6)	0.0092 (5)	0.0021 (5)	0.0019 (5)
N2	0.0250 (7)	0.0214 (6)	0.0236 (6)	0.0100 (5)	0.0049 (5)	0.0066 (5)
N3	0.0199 (6)	0.0210 (6)	0.0245 (6)	0.0094 (5)	0.0017 (5)	0.0024 (5)
N4	0.0272 (7)	0.0284 (7)	0.0207 (6)	0.0151 (6)	−0.0016 (5)	0.0002 (5)
C1	0.0240 (7)	0.0245 (7)	0.0220 (7)	0.0117 (6)	0.0050 (6)	0.0037 (6)
C2	0.0200 (7)	0.0194 (7)	0.0188 (7)	0.0069 (6)	−0.0004 (5)	0.0010 (5)
C3	0.0223 (7)	0.0182 (7)	0.0221 (7)	0.0094 (6)	−0.0032 (6)	−0.0008 (5)
C4	0.0279 (8)	0.0276 (8)	0.0308 (8)	0.0150 (7)	0.0040 (6)	0.0037 (6)
C5	0.0332 (9)	0.0338 (9)	0.0376 (9)	0.0218 (8)	0.0015 (7)	−0.0005 (7)
C6	0.0407 (10)	0.0280 (8)	0.0437 (10)	0.0229 (8)	−0.0037 (8)	0.0016 (7)
C7	0.0354 (9)	0.0239 (8)	0.0362 (9)	0.0148 (7)	−0.0002 (7)	0.0071 (7)
C8	0.0222 (7)	0.0200 (7)	0.0241 (7)	0.0081 (6)	−0.0031 (6)	0.0007 (6)

### Geometric parameters (Å, º)

**Table:**

Cu1—N1	1.9839 (12)	C1—C2	1.492 (2)
Cu1—N3	2.0244 (12)	C1—H1A	0.9900
Cu1—O2	2.5870 (12)	C1—H1B	0.9900
O1—N4	1.2454 (18)	C3—C4	1.393 (2)
O2—N4	1.2621 (17)	C3—C8	1.402 (2)
O3—N4	1.2483 (17)	C4—C5	1.382 (2)
N1—C2	1.3250 (19)	C4—H4	0.9500
N1—C3	1.4016 (19)	C5—C6	1.401 (3)
N2—C2	1.3390 (19)	C5—H5	0.9500
N2—C8	1.381 (2)	C6—C7	1.378 (3)
N2—H2	0.8800	C6—H6	0.9500
N3—C1	1.4798 (19)	C7—C8	1.390 (2)
N3—H3A	0.9200	C7—H7	0.9500
N3—H3B	0.9200		
			
N1—Cu1—N1^i^	179.999 (1)	N3—C1—H1A	110.1
N1—Cu1—N3	83.84 (5)	C2—C1—H1A	110.1
N1^i^—Cu1—N3	96.16 (5)	N3—C1—H1B	110.1
N1—Cu1—N3^i^	96.16 (5)	C2—C1—H1B	110.1
N1^i^—Cu1—N3^i^	83.84 (5)	H1A—C1—H1B	108.4
N3—Cu1—N3^i^	180.00 (5)	N1—C2—N2	112.60 (13)
N1—Cu1—O2	94.86 (4)	N1—C2—C1	121.64 (13)
N1^i^—Cu1—O2	85.14 (4)	N2—C2—C1	125.70 (13)
N3—Cu1—O2	99.29 (5)	C4—C3—N1	132.16 (14)
N3^i^—Cu1—O2	80.71 (5)	C4—C3—C8	119.72 (14)
N4—O2—Cu1	118.50 (9)	N1—C3—C8	108.09 (13)
C2—N1—C3	105.70 (12)	C5—C4—C3	117.45 (15)
C2—N1—Cu1	112.29 (10)	C5—C4—H4	121.3
C3—N1—Cu1	141.90 (10)	C3—C4—H4	121.3
C2—N2—C8	107.67 (12)	C4—C5—C6	122.28 (16)
C2—N2—H2	126.2	C4—C5—H5	118.9
C8—N2—H2	126.2	C6—C5—H5	118.9
C1—N3—Cu1	111.94 (9)	C7—C6—C5	120.84 (15)
C1—N3—H3A	109.2	C7—C6—H6	119.6
Cu1—N3—H3A	109.2	C5—C6—H6	119.6
C1—N3—H3B	109.2	C6—C7—C8	116.89 (15)
Cu1—N3—H3B	109.2	C6—C7—H7	121.6
H3A—N3—H3B	107.9	C8—C7—H7	121.6
O1—N4—O3	120.07 (13)	N2—C8—C7	131.30 (15)
O1—N4—O2	120.38 (14)	N2—C8—C3	105.93 (13)
O3—N4—O2	119.55 (13)	C7—C8—C3	122.77 (15)
N3—C1—C2	107.95 (12)		

Symmetry code: (i) −*x*+5/3, −*y*+1/3, −*z*+1/3.

### Hydrogen-bond geometry (Å, º)

**Table:**

*D*—H···*A*	*D*—H	H···*A*	*D*···*A*	*D*—H···*A*
N3—H3*A*···O1^ii^	0.92	2.42	3.266 (2)	153
N3—H3*A*···O3^ii^	0.92	2.37	3.0521 (17)	131
N3—H3*B*···O1^i^	0.92	2.33	3.1036 (19)	142
N2—H2···O3^iii^	0.88	2.50	3.0276 (18)	119
N2—H2···O3^iv^	0.88	2.40	3.0823 (18)	134
N2—H2···O2^iii^	0.88	2.20	2.8901 (17)	135

Symmetry codes: (i) −*x*+5/3, −*y*+1/3, −*z*+1/3; (ii) −*y*+4/3, *x*−*y*−1/3, *z*−1/3; (iii) −*x*+*y*+5/3, −*x*+4/3, *z*−2/3; (iv) *x*, *y*, *z*−1.

## References

[bb1] Bruker (2007). *APEX2* and *SAINT* Bruker AXS Inc., Madison, Wisconsin, USA.

[bb2] Gable, R. W., Hartshorn, R. M., Mcfadyen, W. D. & Nunno, L. (1996). *Aust. J. Chem.* **49**, 625–632.

[bb3] Gómez-Segura, J., Prieto, M. J., Font-Bardia, M., Solans, X. & Moreno, V. (2006). *Inorg. Chem.* **45**, 10031–10033. 10.1021/ic061292t 17140202

[bb4] He, Y., Kou, H.-Z., Wang, R.-J. & Li, Y.-D. (2003). *Transition Met. Chem.* **28**, 464–467.

[bb5] Jiang, Y.-B., Kou, H.-Z., Gao, F. & Wang, R.-J. (2004). *Acta Cryst.* C**60**, m261–m262. 10.1107/S0108270104009102 15178843

[bb6] Pascaly, M., Duda, M., Schweppe, F., Zurlinden, K., Müller, F. K. & Krebs, B. (2001). *J. Chem. Soc. Dalton Trans.* pp. 828–837.

[bb7] Sheldrick, G. M. (1996). *SADABS* University of Göttingen, Germany.

[bb8] Sheldrick, G. M. (2008). *Acta Cryst.* A**64**, 112–122. 10.1107/S0108767307043930 18156677

